# Circulating tumor DNA to anticipate loco-regional recurrence in early-stage breast cancer: a proof-of-concept study

**DOI:** 10.3389/fonc.2025.1621322

**Published:** 2025-09-11

**Authors:** Valentina Appierto, Elena Tamborini, Paola Tiberio, Adele Busico, Loris De Cecco, Marco Silvestri, Cinzia De Marco, Elena Cavadini, Maria Carmen De Santis, Secondo Folli, Gianfranco Scaperrotta, Rebecca Manitto, Andrea Vingiani, Giancarlo Pruneri, Serena Di Cosimo

**Affiliations:** ^1^ Department of Advanced Diagnostics, Fondazione Istituto di Ricovero e Cura a Carattere Scientifico (IRCCS) Istituto Nazionale dei Tumori, Milano, Italy; ^2^ Medical Oncology and Hematology Unit, IRCCS Humanitas Research, Milan, Italy; ^3^ Department of Experimental Oncology, Fondazione Istituto di Ricovero e Cura a Carattere Scientifico (IRCCS) Istituto Nazionale dei Tumori, Milano, Italy; ^4^ Isinnova s.r.l, Brescia, Italy; ^5^ Breast Unit, Fondazione Istituto di Ricovero e Cura a Carattere Scientifico (IRCCS) Istituto Nazionale dei Tumori, Milano, Italy; ^6^ Radiation Oncology 1, Fondazione Istituto di Ricovero e Cura a Carattere Scientifico (IRCCS) Istituto Nazionale dei Tumori, Milano, Italy; ^7^ Breast Imaging Unit, Fondazione Istituto di Ricovero e Cura a Carattere Scientifico (IRCCS) Istituto Nazionale dei Tumori, Milano, Italy; ^8^ Bioinformatics and Biostatistics Unit, Fondazione Istituto di Ricovero e Cura a Carattere Scientifico (IRCCS) Istituto Nazionale dei Tumori, Milano, Italy

**Keywords:** circulating tumor DNA, loco-regional recurrence (LRR), early breast cancer (EBC), follow-up, next generation sequencing (NGS), digital PCR (dPCR), somatic mutation

## Abstract

**Background:**

Loco-regional recurrence (LRR) poses a clinical challenge for the follow-up of patients treated with curative intent for early-stage breast cancer (EBC). While circulating tumor DNA (ctDNA) has been shown to predict distant metastases, its value for LRR is less characterized.

**Methods:**

Starting from an index case with documented LRR and available tumor and plasma samples, we report the analysis of the prospective phase III fenretinide prevention trial, which primarily aimed to assess the incidence of second malignancy in women with T1-T2 N0 EBC. Patients were eligible if they had FFPE and/or frozen tissue from primary or recurrent invasive tumor for next generation sequencing, and at least three serial plasma samples for ctDNA analysis by digital PCR.

**Results:**

The *TP53* R196* mutation was identified in the primary tumor of the index case with a variant allele frequency (VAF) of 29%, and in the LRR with a VAF of 58%. The same mutation was also detected in plasma prior to both the primary and LRR surgeries with VAFs of 0.19% and 0.12%, respectively. Following treatment, the mutation became undetectable in plasma samples during follow-up, consistent with the absence of recurrence. Among 40 eligible patients from the fenretinide prevention trial, 27 (67.5%) had primary tumor somatic variants trackable in plasma. Median age was 55 years (range, 35-78); stage I (16, 59%) and stage II (11, 41%); mostly luminal-like (19, 70%); median follow-up 173 months (range, 98-193); common mutations included *PIK3CA* (50%), *TP53* (30.7%), and *PTEN* (5.9%). Six patients developed LRR as first event; 4 distant metastases. In all LRR cases, except one, ctDNA was detected prior to surgery and anticipated the clinical diagnosis up to 28 months. Three patients with LRR developed distant metastases 1 to 2 years later.

**Conclusion:**

These findings show the potential of ctDNA for the early detection of LRR in EBC, and its promise as a tool for timely interventions and personalized surveillance strategies.

## Introduction

Breast cancer accounts for nearly a quarter of all female malignancy and represents the leading cause of cancer-related mortality among women worldwide (1). Although substantial advances in early diagnosis and treatment have contributed to ameliorate survival, disease recurrence remains a major clinical challenge.

Loco-regional recurrence (LRR) occurs in approximately 5-15% of patients with early-stage disease and is associated with an increased risk of subsequent systemic spread (2–4). Since LRR is strongly associated with disease-specific mortality (5, 6), its early identification is crucial to improving patient outcomes. However, current surveillance protocols relying on physical examinations and imaging have limited sensitivity in detecting subclinical disease, highlighting the need for more dynamic and specific biomarkers.

Circulating tumor DNA (ctDNA), a component of cell-free DNA shed by tumor cells, has emerged as a promising and minimally invasive biomarker, providing a unique source to monitor disease in real time (7).

In advanced breast cancer, ctDNA is increasingly integrated into clinical decision-making to identify actionable mutations for targeted therapy (8). As an example, plasma *PIK3CA* mutations can guide the use of PI3K inhibitors in patients with hormone receptor-positive/HER2-negative disease; while *ESR1* mutations inform resistance to aromatase inhibitors and the use of novel oral SERDs (9).

In the early-stage breast cancer (EBC), ctDNA is technically more challenging due to its lower levels. Nevertheless, ctDNA has shown clinical potential for prognosis, prediction of pathological complete response after neoadjuvant therapy, detection of residual disease after surgery, and early identification of relapse during follow-up (10).

Plasma serial monitoring for individual tumor mutations in *TP53, PIK3CA, GATA3, ARID1A, AKT* which are the most commonly found altered genes in breast cancer (11–13), has shown to detect minimal residual disease even months before clinical or radiologic evidence of overt metastases (14–16). These studies have reported the potential of ctDNA in predicting distant recurrence in operable breast cancer, with encouraging results in terms of sensitivity and specificity and supported prospective trials that aim to assess the utility of ctDNA for EBC (17–19). Nonetheless, most ctDNA research has focused on distant recurrence or treatment monitoring, whereas data on the use of ctDNA for detecting LRR, particularly in early-stage disease, remain limited.

In this study, we explored the role of ctDNA for the early identification of LRR in EBC patients. As a first step, we retrospectively analyzed ctDNA dynamics in an index case with a documented LRR and available tumor and plasma samples. Building on this observation, we report a *post hoc* analysis of a prospective phase III prevention trial enrolling patients with surgically treated for stage I-II breast cancer (20), for whom serial plasma samples were prospectively collected during follow-up. Our goal was to evaluate whether ctDNA monitoring could anticipate LRR, thus envisaging its integration into tailored post-treatment surveillance protocols.

## Materials and methods

### Study design and patient population

This study was based on a single breast cancer patient, referred to as the index case, and evaluable EBC patients from the prospective phase III fenretinide prevention trial (20).

For the index case, clinico-pathological data, sequencing of tumor tissue and profiling of plasma samples were evaluated at diagnosis, at the time of LRR, and during follow-up.

Patients from the prospective phase III fenretinide prevention trial were eligible if they fulfilled the following criteria: i) FFPE or frozen tumor tissue suitable for somatic single-nucleotide variants (SNV) identification by next generation sequencing (NGS), and ii) at least three plasma samples prospectively collected during follow-up. It is worth noting that the trial included women aged 30–70 years with T1-T2 N0 breast cancer who were treated with surgery ± radiotherapy, without adjuvant systemic chemo- or endocrine-therapy. Participants underwent semi-annual clinical evaluations, annual mammography and chest X-rays, and biennial bone scans. During trial follow-up serial plasma samples were collected every 6 months until relapse. These samples had been previously used for ancillary studies (reviewed in 21). Written informed consent and Institutional Review Board approval were obtained for the original and current analyses. As original assessment did not include HER2 status, HER2 was evaluated for the purpose of the current analysis by immunohistochemistry (IHC) and *in situ* hybridization as per standard practice (22).

### Tumor tissue DNA extraction and sequencing

DNA was isolated from four sections of primary tumor FFPE tissues (10 μm thick slides with tumor cellularity ≥50%) using the GeneRead DNA FFPE Kit (Qiagen, Valencia, CA, USA) according to the manufacturer instructions. For DNA extraction from frozen samples the QIAamp DNA Mini Kit (Qiagen) was used following the manufacturer protocol. DNA quantity was assessed using Qubit dsDNA HS Assay Kit (Thermo Fisher Scientific, Waltham, MA, USA). Targeted NGS was performed using the Ion AmpliSeq™ Cancer Hotspot Panel v2 (Thermo Fisher Scientific), which includes 207 amplicons covering of 50 oncogenes and tumor suppressor genes. Cases with no detectable somatic variants using this panel and with available matched germline DNA from normal (non-tumoral) lymph nodes or breast tissue were subsequently analyzed with the Ion AmpliSeq™ Comprehensive Cancer Panel (Thermo Fisher Scientific), which targets all exons of 409 cancer-related genes. Detailed procedures are described in the **Supplementary Materials**.

### Plasma collection and cell-free DNA extraction

For the index case, blood was collected in K2EDTA tubes preoperatively at both surgical time points and during follow-up. For the fenretinide cohort, blood was collected in heparin tubes at baseline, follow-up visits, and until relapse. Plasma was separated by centrifugation and stored at −80°C. cell-free DNA (cfDNA) was extracted using the QIAamp Circulating Nucleic Acid Kit (Qiagen), eluted in 35 µL of AVE buffer, and quantified using Qubit dsDNA HS Assay Kit (Thermo Fisher Scientific). For heparinized samples, eluates were treated with heparinase I (1u/µl) for 1 hour at room temperature (23). Spike-in experiments using a 125 bp Lambda DNA fragment confirmed successful dPCR performance after heparinase treatment (**Supplementary Material**, **Supplementary Figure S1A**).

### digital polymerase chain reaction

dPCR assays (TaqMan SNP Genotyping, Thermo Fisher) were developed to validate somatic variants in tumor tissue and to track them in plasma. When dPCR assays wet-lab validated by the manufacturer were not available custom mutation-specific dPCR assays were designed using the Thermo Fisher Scientific custom SNP genotyping assay tool.

PCR reactions were run on the ProFlex™ 2x Flat PCR System thermal cycler (Thermo Fisher), incubating the chips at 96°C for 10 minutes, followed by 45 cycles of 56°C for 2 minutes, 98°C for 30 seconds and 60°C for 2 minutes. Chips were read on the QuantStudio^®^ 3D Digital PCR Instrument (Thermo Fisher) and analyzed using QuantStudio^®^ 3D AnalysisSuite™ Server (Thermo Fisher). Negative controls were performed with wild type (wt) genome (Promega Corporation, Madison, WI, USA) and no DNA template (NTC) were included in every run.

### Plasma DNA pre-amplification

Plasma DNA was pre-enriched by amplification using TaqMan^®^ PreAmp Master Mix Kit (Thermo Fisher Scientific), as previously described (24). Briefly, sample volume was reduced by Eppendorf Concentrator 5301 (Epperdorf Srl, Milano, Italy) to 14 µl. Pre-amplification reaction was performed in a volume of 10 µl containing 4 µl of DNA template, 5 µl of pre-amplification mastermix, and 1 µl of the same specific primers and probes designed for dPCR (at a final dilution of 0.05x). The amplification reaction was initiated by incubation of samples at 95°C for 10 minutes followed by 12 cycles of 95°C for 15 seconds, 60°C for 4 minutes. The pre-amplified PCR products were then diluted 1:100-1:500 and 7 µl of dilutions were used to perform dPCR.

As negative controls, wild-type genome and NTC, in place of DNA template, were included in each pre-amplification reaction, and assayed by dPCR. VAFs estimated by dPCR with or without pre-amplification showed a strong linear correlation, with an r² = 0.96 (**Supplementary Figure S1B**), indicating that pre-amplification does not impair the evaluation of VAFs.

## Results

### Presentation of the case

A 37-year-old premenopausal woman, with no family history of breast or ovarian cancer, and confirmed germline *BRCA1* and *BRCA2* wild-type, was referred to our institution following a left breast tumorectomy performed at another hospital.

Physical examination revealed a well-healed surgical scar on the left breast and a palpable lymph node approximately 1 cm in diameter in the left axilla. Mammography and ultrasound showed both hyperplastic and suspicious nodes in the left axilla. The patient underwent left upper-outer quadrantectomy and axillary lymph node dissection 5 weeks after the initial surgery.

Histological examination of the definitive surgical specimen showed an invasive breast carcinoma, grade III, measuring 18 mm in greatest dimension (pT1c), with metastatic involvement of 15 out of 30 axillary lymph nodes (pN3). Immunohistochemistry was negative for estrogen receptor (ER), progesterone receptor (PgR), and HER2, consistent with a triple-negative phenotype; Ki-67 proliferation index was 90%.

Adjuvant chemotherapy was initiated with four cycles of doxorubicin and paclitaxel (AT regimen) every 21 days, followed by four cycles of cyclophosphamide, methotrexate, and fluorouracil (CMF) administered on days 1 and 8 every 28 days. The patient completed the treatment without major complications.

Approximately one year after the initial surgery, the patient developed local recurrence at the site of the primary tumor. Imaging studies confirmed the absence of distant metastases. She underwent radical mastectomy. Histological evaluation confirmed a triple-negative LRR, spanning at least 1.7 cm in greatest dimension (rpT1c). Following surgery, the patient received six cycles of carboplatin and gemcitabine administered on days 1 and 8, every 21 days, followed by adjuvant radiotherapy to the chest wall and supraclavicular lymph nodes, for a total dose of 50.4 Gy delivered in 28 fractions of 1.8 Gy per day.

**Figure 1:**
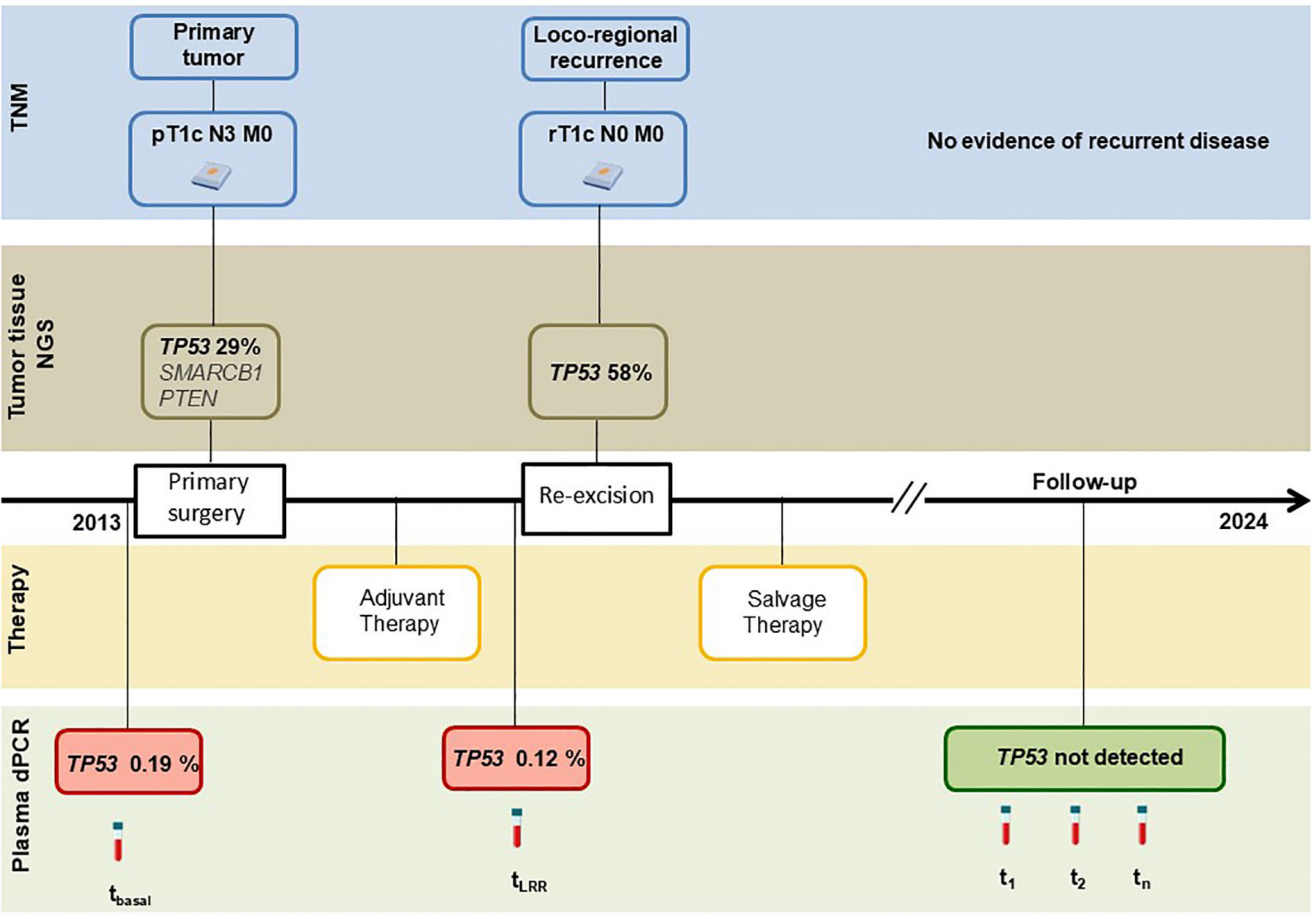
Specimen collection, molecular testing, and clinical timeline for the index case.

The patient has remained under routine surveillance. At her most recent follow-up in June 2024, clinical examination and imaging studies showed no evidence of local, regional, or distant disease recurrence (**Figure 1**).

Tumor targeted NGS identified multiple somatic variants, specifically *TP53* (c.586C>T, p.R196*, VAF 29%), *SMARCB1* (c.215C>A, p.T72K, VAF 38%), and *PTEN* (c.203A>G, p.Y68C, VAF 8%). Among these, only the *TP53* p.R196* mutation was detected in both the primary tumor and the LRR, and was therefore selected for subsequent plasma analysis. *SMARCB1* and *PTEN* mutations were not detected in the recurrent lesion and were not assessed in plasma samples. *TP53* p.R196* mutation was confirmed by dPCR in both lesions, including the recurrent tumor, where it was present at a VAF of 58%. In addition, the same mutation was found in preoperative plasma samples of both primary and LRR surgeries with values of VAF of 0.19% and 0.12%, respectively. Notably, it was undetectable in all five samples prospectively collected during follow-up, when the patient remained disease-free.

### ctDNA monitoring in the trial cohort

**Figure 2:**
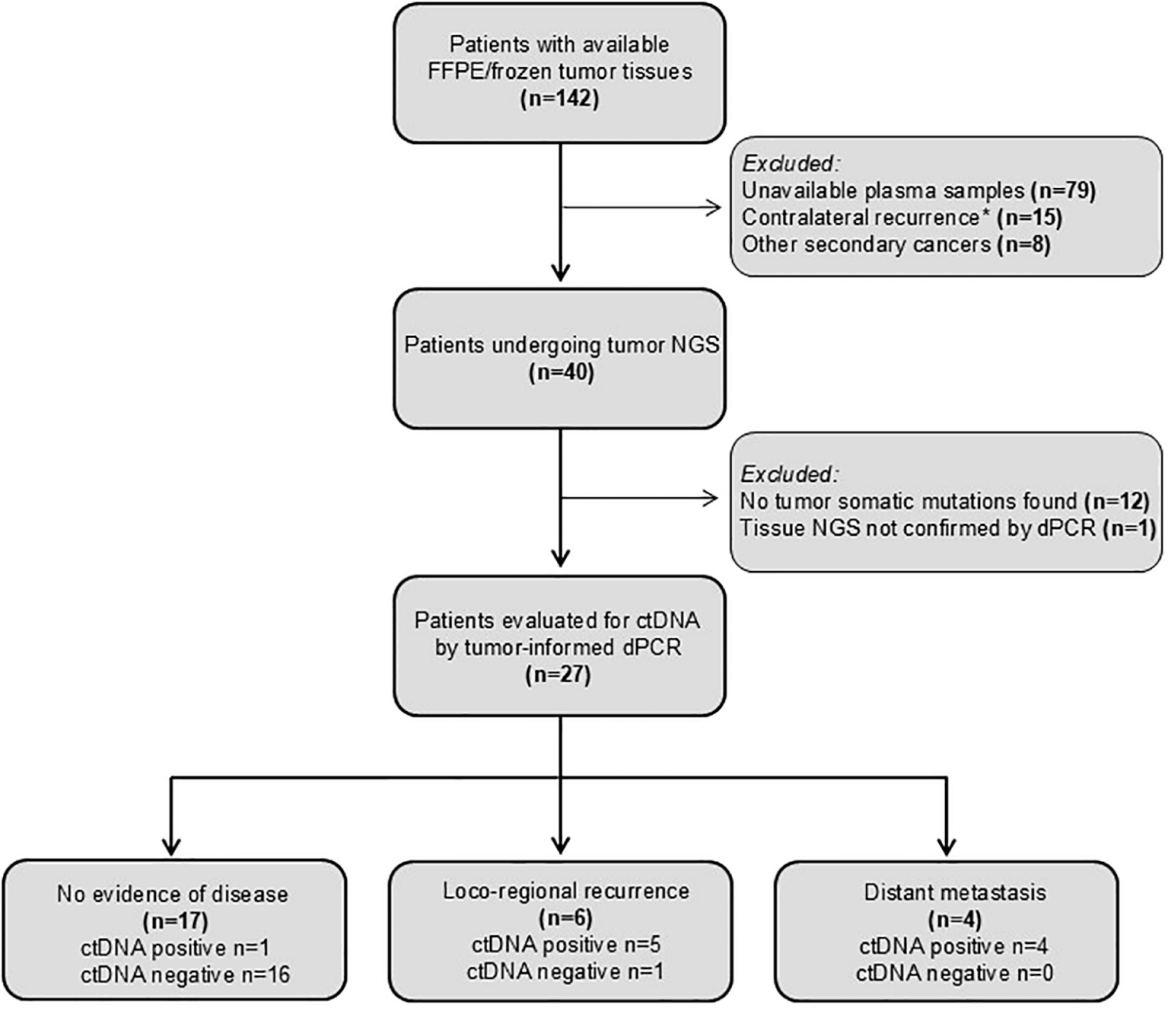
CONSORT diagram showing patients analyzed and reasons for their exclusion. Contralateral recurrences was excluded as it may represent a distinct primary tumor.

Based on these results, we report a *post hoc* analysis of patients enrolled in the prospective phase III fenretinide prevention trial. A total of 40 patients were considered eligible based on the prefixed criteria reported above (**Figure 2**).

Even though DNA was extracted from specimens more than 20 years old, one or more somatic mutations suitable for ctDNA tracking were identified in tumor samples from 27 patients of the 40 analyzed (67.5%), enabling the development of tumor-informed dPCR assays for plasma analysis. Study patients, tumor and treatment characteristics are summarized in **Table 1**.

**Table 1:** Study patient population.

Characteristics	n (%)
Age	
≤ 50 years	9 (33)
> 50 years	18 (67)
Sex	
Female	27 (100)
Male	0
Primary tumor	
≤ 2 cm	15 (56)
> 2 cm	12 (44)
Nodal status	
N0	27
N≥ 1	0
Histology	
Invasive ductal carcinoma	12 (44)
Other	15 (56)
Receptor status	
ER-positive	19 (70)
PgR-positive	13 (48)
HER2	
0	6 (22)
low	15 (56)
3+/amplified	3 (11)
missing	3 (11)
Luminal-like	19 (70)
Triple-negative	5 (19)
HER2-overexpressing	3 (11)
Treatment	
BCT	19 (70)
BCS	4 (15)
Radical mastectomy	4 (15)
Adjuvant therapy	
Fenretinide	23 (85)
*Nihil*	4 (15)
Events	
Loco-regional recurrence	6 (22)
Distant metastases	4 (15)
None	17(63)
Follow-up	
Median (IQR), months	173 (98-193)
Plasma samples per patient	
Median number (range)	4 (3-7)

*ER, Estrogen receptor; PgR, Progesterone receptor; BCT, Breast-Conserving Therapy; BCS, Breast-Conserving Surgery; HER2, Human Epidermal Growth Factor Receptor 2; Luminal-like, ER+ and/or PgR+ without known HER2 overexpression; triple-negative, ER-/PgR-/HER2 negative;* IQR, Interquartile range.

A total of 34 tumor mutations were found by NGS and validated by dPCR: 23 patients had 1 mutation (85%), 3 had 2 (11%), and 1 patient had 3 (4%). The most frequently mutated genes were *PIK3CA* (17/34, 50%), *TP53* (8/34, 23.5%), and *PTEN* (2/34, 5.9%). VAFs estimated by NGS and dPCR showed a strong linear correlation, with an r² = 0.90 (**Supplementary Figure S1C**). Details on validated mutations are provided in **Supplementary Table S1**.

**Figure 3:**
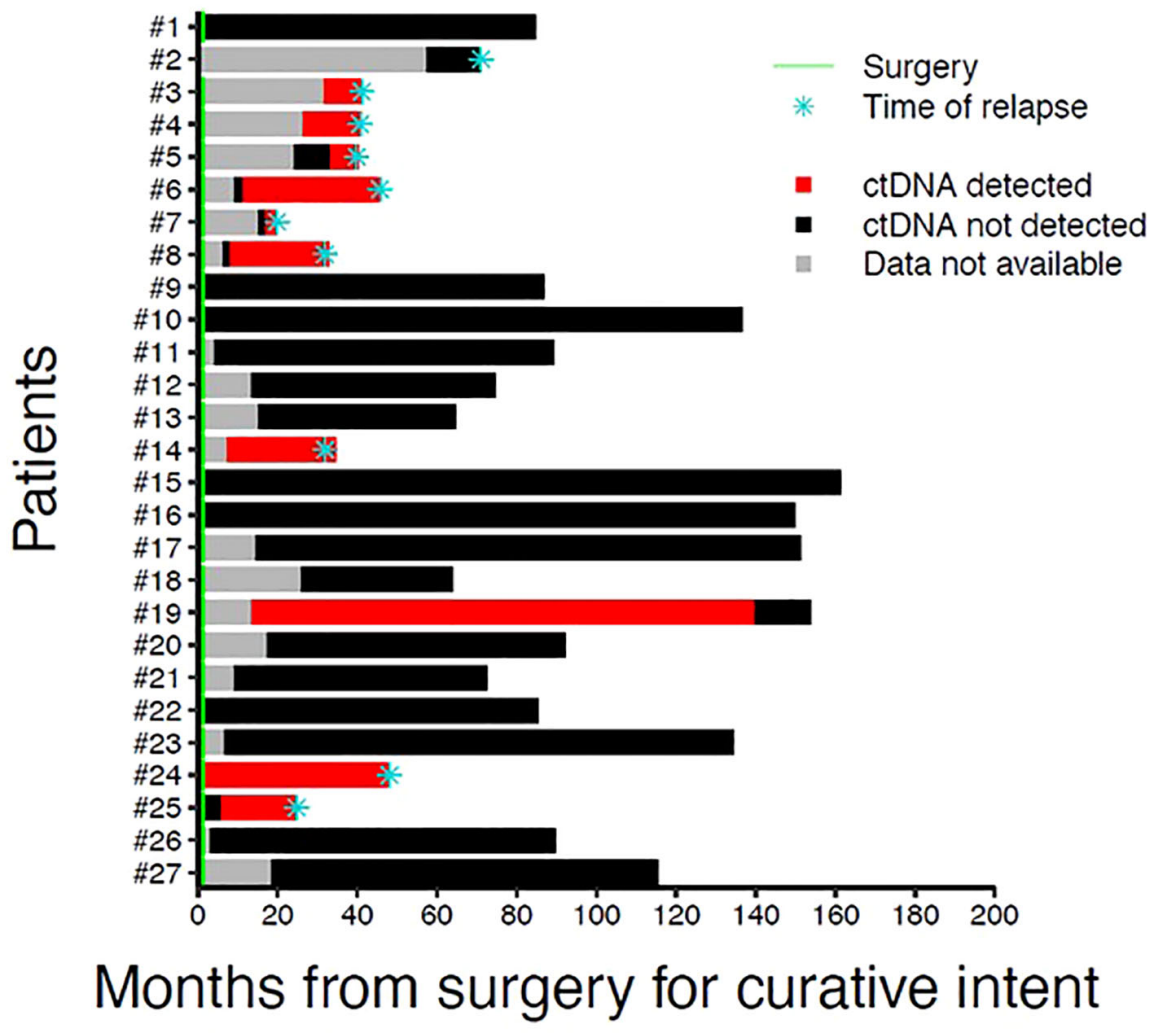
Swimmer plot representing longitudinal ctDNA tracking for study patients. For each patient, times of surgical resection and relapse are indicated by a green line and a blue asterisk, respectively.

**Figure 4:**
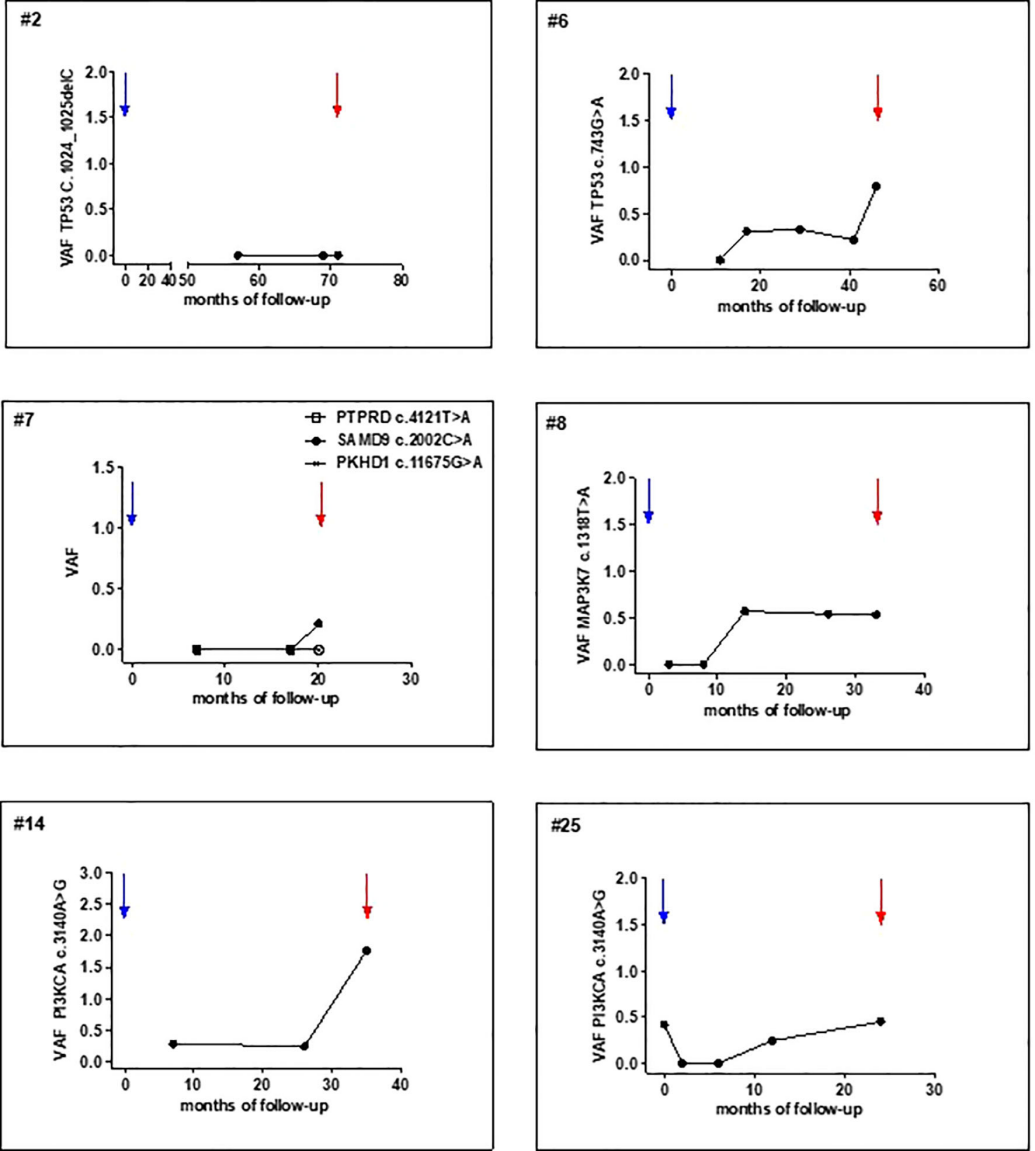
Post-operative tumor mutation tracking in plasma samples of patients surgically treated for primary T1-T2 N0. x-axis, time of follow-up (months from primary surgery); y-axis, mutation VAF (%). Blue and red arrows indicate primary tumor resection and clinical detection of LRR, respectively.

A total of 112 plasma samples were analyzed for the identified tumor mutations by dPCR. Among the recurrent patients, 6 with LRR and 4 with distant metastases, ctDNA was detectable at the time of clinical diagnosis in all the cases except one with VAF values ranging from 0.113 to 4.69 (**Figures 3**, **4**, **Supplementary Figure S2**). Detection of ctDNA prior to clinical diagnosis was observed in both LRR and distant relapses with a median lead time of 24.5 months (IQR: 17.9 - 27.9) for LRR and 12.25 months (IQR: 7.4 - 22.8) for metastatic disease compared with clinical relapse. In patients #2, #7, and #8, LRR was eventually followed by metastasis (**Supplementary Table S2**). As the protocol stopped plasma sampling after the initial event, ctDNA monitoring until progression was precluded.

In 17 patients without evidence of clinical recurrence, ctDNA was undetectable in all 67 samples except in patient #19 that showed ctDNA in three consecutive samples which turned negative in the last sampling (**Supplementary Figure S3**). No documented clinical breast relapse was available. The patient died 52 days after last blood draw, at the age of 79 years.

Even considering the limited number of cases, ctDNA showed an overall accuracy of 92% (95% CI: 75-99), with a sensitivity of 90% (95% CI: 55-99), and specificity of 94% (95% CI: 71-99) (**Supplementary Table S3**).

## Discussion

Tumor-specific mutations in post-operative plasma samples of patients with EBC may serve as a monitoring tool for detecting LRR. In the index case, a *TP53* mutation shared by primary and LRR was detected in plasma at diagnosis and at the time of relapse but remained undetectable during follow-up consistent with the patient remaining disease-free. In a prevention trial cohort, ctDNA not only identified LRR, but also anticipated its clinical diagnosis.

These findings expand the evidence supporting the potential applications of ctDNA for breast cancer management, complementing its established role in predicting distant recurrence (14). While prior research focused on ctDNA for monitoring advanced disease or therapy response, recent advances suggest we should reconsider the full potential of ctDNA. Beyond simply detecting recurrence, ctDNA can assess surgical efficacy, identify residual disease, and monitor evolution during remission (25, 26), as a dynamic biomarker guiding personalized care across the treatment continuum. Our earlier study detecting primary tumor mutations in blood from patients with ductal carcinoma *in situ* (27) further reinforced the potential of ctDNA analysis across all breast cancer stages, from pre-invasive to recurrent disease.

Early diagnosis of LRR in breast cancer remains a cornerstone of post-treatment management, given its critical implications for prognosis and therapeutic strategy. LRR not only signals potential treatment failure but may also precede distant metastases, thereby influencing both disease-free and overall survival (4, 28). Traditional mammographic surveillance after breast-conserving therapy aims to detect ipsilateral recurrences and contralateral breast cancer, which occur with an annual risk of 0.2-2% and 0.4%, respectively (1).

While annual diagnostic mammography is commonly performed during the first three to five years to identify residual or recurrent disease and to establish a reliable post-treatment baseline (29), high-quality evidence regarding its optimal frequency, methodology, and survival benefit remains limited (30, 31). Nonetheless, retrospective studies suggest a survival advantage for mammographically detected recurrences (32), despite the lower sensitivity and specificity observed in women with a personal history of breast cancer (33).

Hence, ctDNA holds the potential to assist the diagnosis of LRR still at a potentially curable status by integrating both imaging and molecular tools to optimize early detection and management of LRR in breast cancer survivors.

In addition, our observation that ctDNA-negative patients remained recurrence-free supports the personalization of imaging assessment based on individual risk. We have to recognize that one patient (out of 17) had detectable ctDNA without developing overt recurrence during the follow-up period. Although patient #19 had a false positive result, given the absence of recurrence and subsequent ctDNA clearance, this was the only such case in the study population, with ctDNA demonstrating a high positive predictive value of 90% (95% CI: 57-98%) and a similarly high negative predictive value of 94% (95% CI: 73-99%). The patient died 52 days after the last blood draw. Unfortunately, attempts to gather further clinical information on the cause of death through possible means were unsuccessful. Therefore, we cannot exclude the possibilities of an undiagnosed malignancy, a subclinical disease, or other unrelated causes. These findings underscore the importance of maintaining high specificity in the development of new ctDNA assays, both to prevent unnecessary psychological distress due to false positive results and to address technical challenges such as background signals potentially related to ineffective erythropoiesis (34).

It is worth noting that, in current clinical practice, international guidelines recommend a rational use of NGS in advanced breast cancer, prioritizing targeted testing for known actionable mutations (e.g., PIK3CA, BRCA1/2, ESR1, etc.). Conversely, the assessment of minimal residual disease in the post-operative setting for localized disease is not yet standard practice and should be limited to patients enrolled in clinical research protocols.

Studies have shown that ctDNA detection after surgery can predict early relapse and a worse prognosis in breast cancer, with a median lead time of 7.9 to 18.9 months before clinical recurrence (reviewed in 35). Most of these studies assess ctDNA using a tumor-informed approach, often through digital PCR, as in our case, or with assays such as Signatera, which offer high sensitivity with a limit of detection 0.01%. More recently, novel methods for ctDNA analysis have emerged. Invitae PCM tracks 18–50 tumor-specific variants and detected ctDNA in 10 of 13 patients who experienced relapse, with a median lead time of 13.7 months and no false positives among patients who did not relapse. NeXT Personal combines whole-genome sequencing-based tumor-informed panels with a fixed panel of clinically relevant variants, allowing the tracking of up to 1,800 tumor-specific mutations with a level of detection as low as 1 part per million, and a reported lead time for relapse of 11.7 months (36).

Our study contributes to this expanding field by specifically focusing the potential of ctDNA to predict loco-regional recurrence, an aspect that has received limited attention to date. The strength of our work lies in the use of a well-defined patient population enrolled in a prospective clinical trial designed to evaluate loco-regional relapse, offering a robust framework for analyzing ctDNA dynamics in this setting. Moreover, our findings strengthen the case for using ctDNA to detect disease early and to initiate treatment sooner, particularly when the recurrence is still localized and therefore amenable to treatment with curative intent. While the findings of this study advance the understanding of the potential of ctDNA in LRR detection, several limitations should be acknowledged. The study sample size was relatively small, the use of historical tissue and plasma samples may affect the applicability of our results to a contemporary clinical setting, and the variable timing for post-surgical blood drawings could affect the evaluation of relapse/progression anticipation. In addition, the extraction of DNA from frozen recurrent tissue specimens in five cases represents a technical limitation. However, as direct plasma sequencing technologies continue to evolve and increase in level of detection, tumor-informed approaches may eventually be complemented, or even replaced, by direct, tumor agnostic ctDNA profiling, particularly in settings where tissue is unavailable or archival material is suboptimal. Future investigations should include prospective clinical trials to determine whether ctDNA-guided interventions improve patient management. Key questions remain about optimal sampling frequency and the clinical utility of quantitative ctDNA monitoring over time.

## Conclusions

Our findings suggest that ctDNA represents a promising tool for the detection of LRR following curative treatment of EBC. The strengths of this study include the use of highly sensitive, tumor-informed digital PCR technology; the availability of samples from a cohort enrolled in a study specifically designed to monitor second primary breast cancers; and the presence of multiple prospectively collected longitudinal plasma samples. Furthermore, ctDNA analysis was conducted retrospectively in patients who were regularly monitored with breast imaging as part of the study protocol, thereby minimizing the confounding bias that has historically affected ctDNA studies in metastatic settings, where imaging was often irregular. Nonetheless, several limitations must be acknowledged, particularly the detection of VAFs frequently below 0.2%, highlighting the need to enhance the sensitivity and specificity of direct profiling assays to render ctDNA assessment sufficiently practical for wide routine clinical application. Further confirmation by independent studies will be essential to corroborate these findings and support the integration of ctDNA analysis into the multidisciplinary management of patients treated for early-stage breast cancer.

## Data Availability

All data supporting the findings of this study, including somatic variants, are reported in the manuscript and its **Supplementary Material**. Further inquiries and additional details may be directed to the corresponding author.
