# Supplementary material for: Circulating tumor DNA to anticipate loco-regional recurrence in early-stage breast cancer: a proof-of-concept study
Source: Front Oncol. 2025 Sep 11;15:1621322. doi: 10.3389/fonc.2025.1621322 (PMC12460102; doi:10.3389/fonc.2025.1621322)
Supplement: Supplementary file 1 [file DataSheet1.pdf]

Supplementary Table S1

Identification and validation of tumor variants

Pt ID	PANEL	GENE	CHR	NUCLEOTIDE CHANGE	PROTEIN	% VAF
#1	HS	*PI3KCA*	3	c.1035T>A	p.N345K	26
#2	HS	*TP53*	17	c.1024_1025delC	p.R342fs	23
#3	HS	*PI3KCA*	3	c.2681T>C	p.F894L	10
#4	HS	*ALK*	2	c.3743+594T>C	intronic	51
#5	HS	*PTEN*	10	c.79+5675A>T	intronic	16
#6	HS	*TP53*	17	c.743G>A	p.R248Q	67
#7	CCP	*PTPRD*	9	c.4121T>A	p.V1374E	11
		SAMD9	7	c.2002C>A	p.P668H	14
		PKHD1	6	c.11675G>A	R3892Q	14
#8	CCP	*MAP3K7*	6	c.1318T>A	p.C440S	8
#9	HS	*PI3KCA*	3	c.1633G>A	p.E545K	38
#10	HS	*TP53*	17	c.849_850delG	p.E283fs	36
		ERBB2	17	c.2329G>T	p.E777*	51
#11	HS	*TP53*	17	c.637C>A	p.Q213K	21
#12	HS	*PI3KCA*	3	c.1035T>A	p.N345K	37
#13	HS	*PI3KCA*	3	c.1633G>A	p.E545K	24
#14	HS	*PI3KCA*	3	c.3140A>G	p.H1047R	7
#15	HS	*PI3KCA*	3	c.1035T>A	p.N345K	27
		PI3KCA	3	c.3207A>G	p.K1069=	24
#16	HS	*TP53*	17	c.658A>C	p.I220L	18
#17	HS	*PI3KCA*	3	c.1624G>A	p.E542K	23
#18	HS	*PI3KCA*	3	c.3140A>G	p.H1047R	32
#19	HS	*TP53*	17	c.574G>A	p.G192S	32
	HS	*PI3KCA*	3	c.3140A>G	p.H1047R	22
#20	HS	*PI3KCA*	3	c.1035T>A	p.N345K	23
#21	HS	*TP53*	17	c.661C>A	p.P221T	30
#22	HS	*PI3KCA*	3	c.3140A>G	p.H1047R	32
		TP53	17	c.309G>T	p.K103N	20
#23	HS	*PI3KCA*	3	c.3140A>G	p.H1047R	43
#24	HS	*PI3KCA*	3	c.1633G>A	p.E545K	31
		PI3KCA	3	c.3130A>T	p.N1044Y	19
#25	HS	*PI3KCA*	3	c.3140A>G	p.H1047R	16
#26	HS	*PTEN*	10	c.976G>C	p.A326P	12
#27	HS	*HNF1A*	12	c.775G>A	p.V259I	3
