# Supplementary material for: Circulating tumor DNA to anticipate loco-regional recurrence in early-stage breast cancer: a proof-of-concept study
Source: Front Oncol. 2025 Sep 11;15:1621322. doi: 10.3389/fonc.2025.1621322 (PMC12460102; doi:10.3389/fonc.2025.1621322)
Supplement: Supplementary file 2 [file DataSheet2.pdf]

Supplementary Table S2. Clinico-pathologic characteristics of the patients eligible for ctDNA analysis

Patient ID	Age at initial diagnosis	TX	Fenretinide	Diagnosis	Tumor size (mm)	ER	PgR	HER2	Breast cancer events (months from surgery)	Time from 1st diagnosis to last follow-up/death* (months)
1	55	QUA	-	IDC+ILC	19	+	-	2+ (not evaluable)	None	183
2	35	QUA+RT	Y	ILC	10	+	-	3+	Local (71)<br>Distant (liver; 83)	96*
3	61	TA+RT	Y	IDC	12	+	+	2+ (not evaluable)	Distant (lung; 41)	80*
4	55	QUA+RT	Y	ILC	30	+	-	1+	Distant (bones; 41)	81*
5	45	QUA	Y	ILC	25	+	+	0	Distant (multiple; 40)	89*
6	54	QUA+RT	Y	IMC	25	-	-	0	Local (45)	235
7	37	QUA+RT	Y	ILC+DCIS+LCIS	9	-	-	0	Local (19)<br>Distant (soft tissue; 31)	72*
8	58	QUA	Y	IDC	25	-	-	3+	Local (33)<br>Distant (soft tissue; 45)	98*
9	62	QUA	Y	IDC	13	+	-	1+	None	189
10	69	QUA+RT	Y	IDC	15	+	+	1+	None	245
11	66	QUA+RT	Y	IDC	18	+	-	1+	None	193
12	53	QUA+RT	Y	IDC	21	+	+	2+ (not amplified)	None	173
13	49	QUA+RT	Y	IDC	17	+	+	1+	None	131
14	55	QUA+RT	Y	IDC	14	+	+	2+ (not amplified)	Local (35); Local (59)	100*
15	60	QUA+RT	Y	IDC	13	+	+	1+	None	183*
16	78	QUA+RT	Y	IMC	24	-	-	0	None	187
17	43	QUA+RT	Y	ILC+LCIS	10	+	+	1+	None	169
18	41	TA+RT	Y	IDC	21	+	+	2+ (not amplified)	None	198
19	67	MA	-	IDC+ILC	21	-	-	2+ (not evaluable)	None	157*
20	47	MA	Y	IDC+ILC	22	+	+	1+	None	147
21	56	MA	-	IDC	23	-	-	3+	None	239
22	63	QUA+RT	-	ILC	22	+	-	1+	None	131
23	38	MA	Y	ILC+IDC	20	+	+	1+	None	193
24	48	QUA+RT	Y	IDC	12	+	+	1+	Distant (bones+lung; 48)	60*
25	41	QUA+RT	Y	ILC	12	+	+	1+	Local (25)	192
26	61	QUA+RT	Y	ILC+IDC	20	-	-	0	None	184
27	55	QUA+RT	Y	IDC+DCIS	25	-	-	0	None	214

TX: Treatment; QUA: quadrantectomy + axillary dissection; MA: mastectomy + axillary dissection; TA: tumorectomy + axillary dissection; RT: radiotherapy; IDC: invasive ductal carcinoma; ILC: invasive lobular carcinoma; DCIS: ductal carcinoma in situ; LCIS: lobular carcinoma in situ; IMC: invasive medullary carcinoma; ER: Estrogen Receptors; PgR: Progesterone Receptors.
