# Supplementary material for: Circulating tumor DNA to anticipate loco-regional recurrence in early-stage breast cancer: a proof-of-concept study
Source: Front Oncol. 2025 Sep 11;15:1621322. doi: 10.3389/fonc.2025.1621322 (PMC12460102; doi:10.3389/fonc.2025.1621322)
Supplement: Supplementary file 3 [file DataSheet3.pdf]

Supplementary Table S3.

Diagnostic performance of ctDNA in the study population (n = 27)

	**Value (%)**	**95% Confidence Interval**
**Sensitivity**	90	55% - 100%
**Specificity**	94	71% - 99%
**Positive Predictive Value**	90	57 % - 98%
**Negative Predictive Value**	94	73% - 99%
