# Supplementary material for: Circulating tumor DNA to anticipate loco-regional recurrence in early-stage breast cancer: a proof-of-concept study
Source: Front Oncol. 2025 Sep 11;15:1621322. doi: 10.3389/fonc.2025.1621322 (PMC12460102; doi:10.3389/fonc.2025.1621322)
Supplement: Supplementary file 4 [file DataSheet4.docx]

**Supplementary material**

**Library preparation, deep amplicon sequencing, and variant calling**

Deep sequencing of hotspot regions of 50 genes covering 207 amplicons of 50 oncogenes or [tumor suppressor genes](https://www.sciencedirect.com/topics/medicine-and-dentistry/tumor-suppressor-gene) (*ABL1, EGFR, GNAS, KRAS, PTPN11, AKT1, ERBB2, GNAQ, MET, RB1, ALK, ERBB4, HNF1A, MLH1, RET, APC, EZH2, HRAS, MPL, SMAD4, ATM, FBXW7, IDH1, NOTCH1, SMARCB1, BRAF, FGFR1, JAK2, NPM1, SMO, CDH1, FGFR2, JAK3, NRAS, SRC, CDKN2A, FGFR3, IDH2, PDGFRA, STK11, CSF1R, FLT3, KDR, PIK3CA, TP53, CTNNB1, GNA11, KIT, PTEN, VHL*) contained in the Ion AmpliSeq Cancer Hotspot Panel v2 (Thermo Fisher Scientific) was performed by employing Ion S5 XL System (Thermo Fisher Scientific). In case of lack of identification of somatic mutations by this panel, a larger gene panel of 409 genes was employed (Ion AmpliSeq™ Comprehensive Cancer Panel, Thermo Fisher Scientific; a full list of genes is available at: https://assets.thermofisher.com/TFS-Assets/CSD/Reference-Materials/ion-ampliseq-cancer-panel-gene-list.pdf).

Ten ng or 40 ng of genomic DNA were used  to amplify 50 gene’s hotspot regions using the Ion AmpliSeq Library Kit2.0 (Thermo Fisher) or the 409 all-exon genes contained in the Comprehensive Cancer Panel (Thermo Fisher) according to the manufacturer’s manual (MAN0006735 rev 5.0). Amplicons were ligated to P1 and Barcode adapters using DNA Ligase. Barcoded libraries were purified using AMPure Beads XP (Beckman Coulter, Pasadena, CA) and PCR-amplified for a total of 5 cycles. After a second round of purification with AMPure Beads, the amplified libraries were sized, their quality was assessed using the Agilent BioAnalyzer DNA High Sensitivity kit (Agilent Technologies) and DNA was quantified using the Qubit dsDNA HS kit (Thermo Fisher). In particular, due to the FFPE origin of the DNA, dsDNA fragments with a length range of 50-150 bp were selected for both panels.

Emulsion PCR and sample enrichment were performed using the Ion One touch 2 instrument according to the manufacturer’s instructions. Briefly, an input concentration of DNA library obtained with the first amplification step was added to the emulsion PCR master mix and the Ion sphere particles (ISPs) and a double phase (oil/water) PCR was performed. Then ISPs were recovered and template positive ISPs were enriched using Dynabeads MyOne Streptavidin C1 beads (Thermo Fisher). 316 and 318 chips were used to sequence samples on Ion torrent PGM using ION PGM 200 sequencing kit V2 following the manufacturer’s instructions.

Data from the PGM sequencing were initially processed using the Ion Torrent platform-specific software Torrent Suite to generate sequence reads, alignment of the reads on the reference genome Hg19, trim adapter sequences, filter and remove poor signal-profile reads.

The variant calling from the sequencing data was generated using the Variant Caller plugin. To avoid erroneous base calling, specific filters were applied to plugin: i) an average coverage depth >100x; ii) each strand coverage >20; iii) a variant frequency on each sample >4% (due to the low quality of samples caused by fixation and long storage); and iv) a quality value >30. Filtered variants were visually examined using the Integrative Genomic Viewer (IGV) tool to assess their quality and to confirm the variant presence on both “+” and “-” strands. Variants that were represented on truncated reads were filtered out. For tumor samples analyzed with Comprehensive Cancer Panel the resulting variants were compared and filtered with variants obtained using germinal DNA extracted from a normal tissue sample belonging to the same patients to exclude polymorphisms. Finally, resulting variations were annotated employing the Ensembl Variant Effect Predictor pipeline, the COSMIC database, and the dbSNP database.

**Evaluation of dPCR** **Compatibility of Heparinized Plasma via Spike-in Assay**

To assess the compatibility of heparin-treated plasma with dPCR workflows, DNA spike-in experiments were performed. Specifically, 0.65 pg of HindIII-digested Lambda DNA (Thermo Fisher Scientific) was added to both EDTA- and heparin-treated plasma samples prior to DNA extraction. Following extraction, eluates from heparinized samples were digested with heparinase I (1 U/µL; Sigma-Aldrich, St. Louis, MO, USA) for 1 hour at room temperature. In both sample types, the 125 bp fragment of Lambda DNA was subjected to pre-amplification and dPCR analysis using the following primers and probe: forward primer CAACCTCAAGCCAGAATGCA, reverse primer CGGAGAGATGGGTAAGCACAA, and probe ATCACTGGCTTTTTTG.

**Supplementary figure legends**

**Supplementary figure S1.** A) Assessment of heparinase I-treated plasma DNA suitability for ctDNA analysis. Hind III-digested Lambda DNA was spiked into three EDTA and three heparin plasma samples. Following DNA extraction and heparinase I treatment, a 125 bp fragment of the spike-in DNA was amplified and quantified by dPCR. Comparable copy numbers/µl were observed across samples. Data represent the mean ± S.D. of three independent experiments.
B) Comparison of VAF estimates by dPCR with and without pre-amplification. Somatic tumor mutations tested by dPCR, with or without pre-amplification, showed strong concordance in VAF estimation (r² = 0.96). C) Validation of tumor mutations identified by NGS using dPCR. Somatic mutations detected in tumor tissue by NGS were confirmed in plasma by dPCR with custom-designed assays. VAF estimates from dPCR and NGS showed high concordance (r² = 0.90).

**Supplementary figure S2.** Post-operative tumor mutation tracking in plasma samples of patients surgically treated for primary T1-T2 N0 who developed distant metastases. x-axis, time of follow-up (months from primary surgery); y-axis, mutation VAF (%). Blue and red arrows indicate primary tumor resection and clinical detection of metastases, respectively.

**Supplementary figure S3.** Post-operative tumor mutation tracking in plasma samples of patients surgically treated for primary T1-T2 N0 who remained disease-free during follow-up. x-axis, time of follow-up (months from primary surgery); y-axis, mutation VAF (%). Blue arrows indicate primary tumor resection.
