# Supplementary figures and images for: Circulating tumor DNA to anticipate loco-regional recurrence in early-stage breast cancer: a proof-of-concept study
Source: Front Oncol. 2025 Sep 11;15:1621322. doi: 10.3389/fonc.2025.1621322 (PMC12460102; doi:10.3389/fonc.2025.1621322)

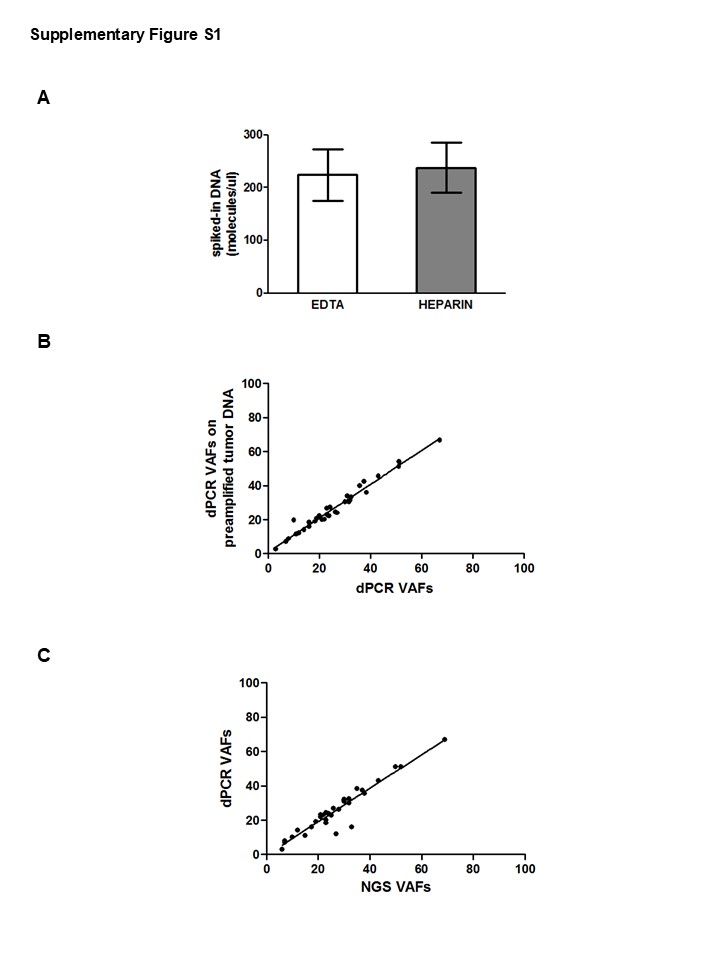

Supplement: Supplementary file 5 [file Image1.jpeg]

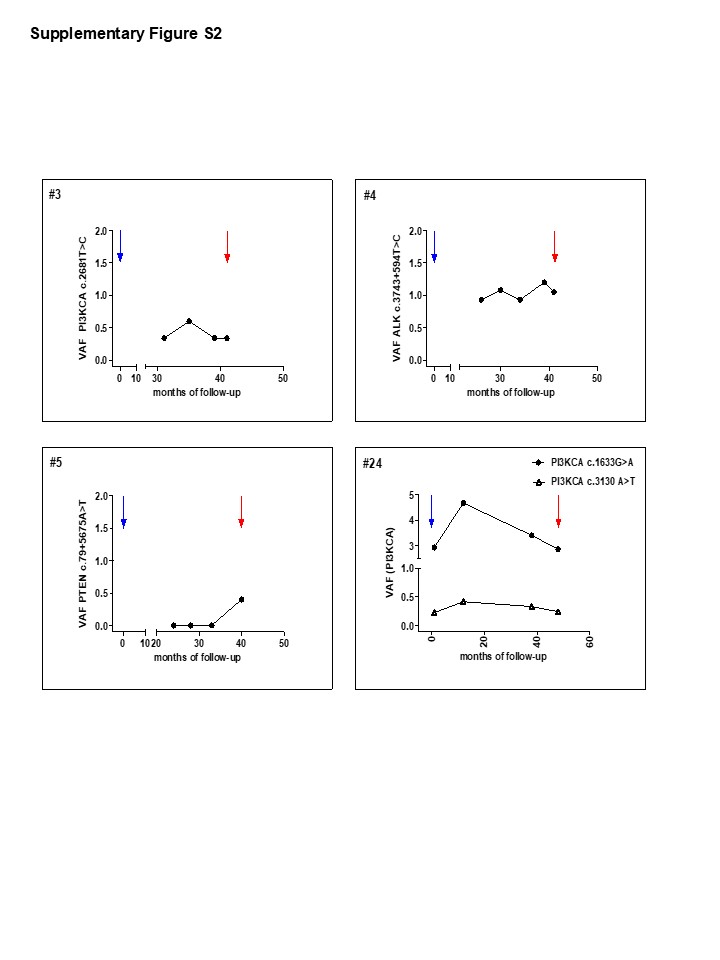

Supplement: Supplementary file 6 [file Image2.jpeg]

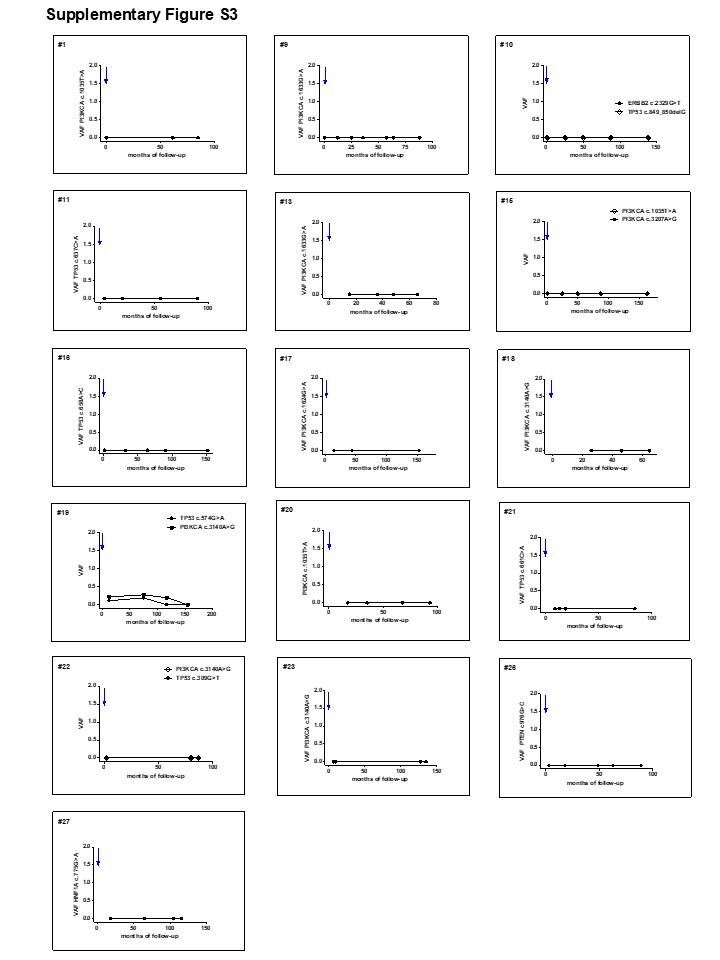

Supplement: Supplementary file 7 [file Image3.jpeg]
